# Associations between m-type phospholipase A2 receptor,human leukocyte antigen gene polymorphisms and idiopathic membranous nephropathy

**DOI:** 10.1080/21655979.2021.1987080

**Published:** 2021-10-29

**Authors:** Xiaogang Liu, Weixi Xu, Chengyuan Yu, Mingao Wang, Ruichan Liu, Rujuan Xie

**Affiliations:** a Department of Nephrology, Shenzhen Hengsheng Hospital, 20 Yintian Rd, Shenzhen, Guangdong, People’s Republic of China; b Department of Nephrology, The 2nd Affiliated of Chengdu Medical College Nuclear Industry 416 Hospital, Chengdu, China; c Department of Geriatric, Shenzhen People’s Hospital (The Second Clinical Medical College, Jinan University; the First Affiliated Hospital, Southern University of Science and Technology), Shenzhen, China; d Department of Nephrology, The First Affiliated Hospital of Harbin Medical University, Harbin, China

**Keywords:** Idiopathic membranous nephropathy, PLA2R, HLA DQA1, single nucleotide polymorphism

## Abstract

Primary membranous nephropathy, also known as idiopathic membranous nephropathy, is an autoimmune disease. As an autoimmune disease, genetic factors are essential in the pathogenesis of IMN. People pay more and more attention to genetics and bioinformatics. With the continuous improvement and development of high-throughput gene sequencing and genotyping technology, it has been confirmed that many genes and their single nucleotide polymorphisms are strongly correlated with IMN disease susceptibility. However, there are few studies on HLA-DQA1 and PLA2R gene polymorphisms and IMN susceptibility in China. The purpose of this study was to investigate whether PLA2R rs2715928 and rs16844715 are related to IMN, the correlation between five SNP loci of PLA2R and HLA-DQA1 and IMN, and the effect of gene-gene interaction among different genotypes of each locus on disease. In this study, 86 patients with IMN confirmed by renal biopsy in the first hospital of Harbin Medical University and 90 healthy controls were selected. All subjects were excluded from secondary membranous nephropathy, pregnant or breastfeeding women, severe primary disease of vital organs, severe infection, major surgery, and severe trauma. Seven selected SNP loci were genotyped using the IMLDR multiple SNP typing kit. Chi-square test and logistic regression were used to analyze the correlation between each SNP and IMN. The general clinical data and laboratory indicators of each subject were recorded, and the relationship between different genotypes and clinical manifestations was analyzed. Among the 7 SNP loci included in the study, except HLA-DQA1 rs2187668, the other 6 loci all met Hardy-Weiberg equilibrium test (P > 0.05). The allele distribution of PLA2R rs2715928 and rs16844715 was significantly different between the IMN group and the healthy control group, and it was closely related to IMN (P < 0.05). There was no statistical difference in the distribution of alleles of rs2715918 between the IMN group and the control group (P* > 0.05), and there was also statistical difference in the distribution of alleles of rs35771982, rs3749117, and rs4664308 between the IMN group and the healthy control group (P < 0.05).The C allele of rs16844715 (OR = 2.03, 95%CI: 1.29–3.19, P* = 0.0140) and the A allele of rs2715928 (OR = 3.18, 95%CI: 1.94–5.24, P* = 3.54E-5), G allele of rs35771982 (OR = 4.07, 95%CI: 2.34–7.08, P* = 4.96E-6), T allele of rs3749117 (OR = 4.07, 95%CI: 2.34–7.08, P* = 4.96E-6), the A allele of rs4664308 (OR = 2.63, 95%CI: 1.54–4.49, P* = 0.0028) was the risk gene of IMN.Through the establishment of different genetic models, we found that,in the additive model, the three SNPs of PLA2R rs2715928 (OR = 5.40, 95%CI: 1.77–16.50, P* = 0.0217) and rs35771982 (OR = 15.15, 95%CI: 2.92–78.48, P* = 0.0084), rs3749117 (OR = 15.15, 95%CI: 2.92–78.48, P* = 0.0084) had a strong correlation with IMN. In the stealth model,homozygous gene risk type of the five SNPs,PLA2R rs16844715 (OR = 2.52, 95%CI: 1.38–4.61, P* = 0.0189) and rs2715928 (OR = 4.30, 95%CI: 2.31–8.03, P* = 3.14E-5), rs35771982 (OR = 4.85, 95%CI: 5.53–9.31, P* = 1.42E-5), rs3749117 (OR = 4.85, 95%CI: 5.53–9.31, P* = 1.42E-5) and rs4664308 (OR = 3.16, 95%CI: 1.67–5.97, P* = 0.0028) had a strong correlation with IMN. The distribution of GT haplotypes and CC haplotypes of rs35771982 and rs3749117 and CA haplotypes and TG haplotypes of rs16844715 and rs4664308 were significantly different between IMN group and control group (P < 0.05). When GMDR software was used to establish a model to analyze the interaction between various SNP sites, it was found that the combination of GG genotype at rs35771982 and AA genotype at rs2715928 was the highest risk of disease. The risk genotypes of rs16844715, rs2715928 and rs4664308 had no effect on the clinical manifestations of IMN (P > 0.05). PLA2R rs2715928 and rs16844715 are associated with susceptibility to IMN. The C allele of rs16844715, the A allele of rs2715928, the G allele of rs35771982, the T allele of rs3749117, and the A allele of rs4664308 are the dangerous genes of IMN. The combination of GG genotype at rs35771982 and AA genotype at rs2715928 poses the greatest risk of disease. Haplotype may affect susceptibility to IMN. The risk genotype had no effect on the clinical manifestations of IMN.

## 1. Introdution

Idiopathic membranous nephropathy (IMN) is one of the most common pathological types of adult primary nephrotic syndrome, characterized by in-situ immune complex deposition in the glomerular subepithelium, diffuse glomerular basal membrane thickening, and the formation of ‘nail processes’ by the fusion of renal podocyte foot processes. The clinical manifestations, course of disease and prognosis vary greatly among different patients [1].

n 1959, Heymann et al. successfully established rat MN model. Subsequently, a podocyte protein called Megalin was identified as the main pathogenic antigen of Heymann’s nephritis. Megalin was expressed in rat glomerular podocytes and proximal renal tubule epithelial cells. However, no Megalin has been found in the glomeruli and podocytes of human IMN patients [2,3]. In recent years, great progress has been made in the study of the pathogenesis of IMN at home and abroad, and a variety of autoantigens have been discovered successively, such as: Neutral endopeptidase (NEP), M-type phospholipase A2 receptor (PLA2R), aldose reductase (AR), superoxide dismutase 2 (SOD2), α-enolase and cationic bovine serum albumin, thrombospondin type-1 domain-containing 7A (THSD7a), and so on. IMN is considered to be an autoimmune-associated disease because IMN is caused by the deposition of immune complexes formed by antigen–antibody binding under the glomerular epithelium.

As an autoimmune disease, genetic factors play an essential role in the pathogenesis of IMN. More and more attention has been paid to genetics and bioinformatics. With the continuous improvement and development of high-throughput gene sequencing and genotyping technology, many genes and their single nucleotide polymorphism (SNP) sites have been proved to be closely related to IMN disease susceptibility. Human major histocompatibility complex (MHC) is also known as human leucocyte antigen (HLA) system. HLA gene complex located in human 6p21.31, is the most polymorphic gene system in human body, HLA-DQA1 belongs to the major histocompatibility complex (MHC) II gene. The main function of MHC class II molecules is to provide antigens derived from extracellular proteins for T cells to recognize [4]. In 1979, British scholars first published reports on the genetic contribution of HLA loci to IMN risk, in which HLA-DR3 was found [5]. The first report on HLA-DQA1 was published by Vaugha et al. [6] in 1989, which found that there was a 4.5kbDNA fragment on the restriction fragment of polymorphism observed in DNA hybridization with HLA-DQA1 probe, which was significantly increased in IMN patients, and this DNA fragment was HLA-DQA1. And it is proposed as a major disease susceptibility factor.

The gene encoding PLA2R is located in the region of human chromosome 2q23~ q24 and contains multiple SNP sites. Mutations in this gene can affect the production of amino acids. For example, the G allele of SNP rs35771982 in exon 5 leads to the change of residues in the second of eight CTLDs, from histidine to aspartic acid. This exposes the PLA2 epitope and stimulates anti-PLA2R antibody formation [7,8]. In 2011, a study involving three separate European cohort populations from France, the Netherlands, and the United Kingdom examined 242,824 common SNPs and identified two important genomic loci associated with IMN: HLA-DQA1 SNP rs2187668 and PLA2R SNP rs4664308.

Subsequently, validation experiments were carried out on a global scale, which not only confirmed a significant association between IMN and the risk alleles of HLA-DQA1 and PLA2R1, but also found gene–gene interactions between the two risk alleles. Several studies from Asia have also found that other SNPs are associated with IMN, and a Korean study found that patients with rs35771982 C/C genotype have a higher susceptibility to IMN and are not associated with secondary MN [7]. The G allele and G/G genotype of rs35771982 may increase the incidence of IMN [8]. A study from Japan found that two novel SNPs (rs2715928 and rs16844715) in PLA2R in addition to SNP rs35771982 were also significantly associated with IMN [9].

Summarizing the above literature, we can find that the results of different studies are different in different regions and different ethnicities, and only a few studies have reported the influence of gene–gene interaction on the pathogenesis and clinical manifestations of IMN. PLA2R rs2715928 is a newly discovered IMN-related SNP in Japan, which has not been detected in Chinese population. In China, there are few studies on the association between PLA2R, HLA-DQA1 gene polymorphism and IMN susceptibility. The variation of PLA2R1 (SNP rs35771982, rs4664308, rs3749117) was found to be a risk factor for IMN in patients with idiopathic membranous nephropathy in South China [10].

In order to investigate whether PLA2R rs2715928 and rs16844715 are associated with IMN, as well as the correlation between PLA2R and HLA-DQA1 SNP loci and IMN, and the influence of gene–gene interaction among various genotypes on disease, five SNPs of PLA2R and HLA-DQA1 gene reported in previous literatures (rs4664308, rs3749117, rs2715918, rs35771982, rs2187668), rs2715928 and rs16844715 were selected as candidate loci for this study to clarify the influence of gene–gene interaction between SNPs on disease susceptibility.

## 2. Materials and methods

### 2.1 Research object

In this study, 86 patients with idiopathic membranous nephropathy who were admitted to the First Department of Nephrology of the First Affiliated Hospital of Harbin Medical University from December 2017 to January 2019 were selected, including 53 males and 33 females, excludes secondary membranous nephropathy caused by systemic disease (systemic lupus erythematosus), diabetes, tumor, hepatitis B, drugs, etc., pregnant or nursing women, severe infection. A total of 90 patients from the Physical Examination Department of the First Affiliated Hospital of Harbin Medical University from December 2017 to January 2019 were selected as the healthy control group, including 35 males and 55 females. Pregnancy or breastfeeding women, urinary diseases, diabetes, hypertension, tumors, infections (such as hepatitis B and C virus, HIV infection) were excluded. No abnormality was found in blood routine, urine routine and renal function examination. This experiment has been approved by the Ethics Committee of the First Affiliated Hospital of Harbin Medical University, and all patients were informed and gave oral consent.

### 2.2 General information and laboratory examination

General information and clinical data of all subjects were collected. Age, gender and height (m) and weight (Kg) of IMN patients were measured before treatment with glucocorticoids or immunosuppressive agents. Fastening vein blood of the patients was collected on the morning after admission, and serum albumin (ALB), serum creatinine (SCR), blood urea nitrogen (BUN), triglyceride (TG) and cholesterol (CHOL) were detected (completed by the biochemical laboratory of our hospital). Twenty-four hours urinary total protein was measured from the next day after admission (completed in the immunology room of our hospital). Body mass index (BMI) was calculated: BMI = weight/height^2^; Blood pressure at rest was measured, measured twice and averaged. SBP and DBP were recorded.

Estimated Glomerular Filtration Rate (eGFR) of the patients was calculated using the CKD-EPI formula, the calculation method is as follows, please refer to Tables 1–1:

**Table 1.** Calculation method of eGFR

Gender	Scr (mg/dl)	Formula
Female	≤0.7	*eGFR = 144X(Scr%0.7)^−0.329^X(0.993)^age^*
	>0.7	*eGFR = 144X(Scr%0.7)-^1.209^X(0.993)^age^*
Male	≤0.9	*eGFR = 144X(Scr%0.7)^−0.311^X(0.993)^age^*
	>0.9	*eGFR = 144X(Scr%0.7)-^1.209^X(0.993)^age^*

Note: The unit of SCR in the formula is mg/dL.

### 2.3 Experimental methods

#### 2.3.1 Experimental instruments and consumable

**Table:** 

Experimental instruments	Company
−80°C refrigerator	China’s haier
Piece head (1000 μl)	Invitrogen U.S.
Ependof tube (1 ml)	Eppendorf Germany
TGUIDE M16 Automatic Nucleic Acid Extraction Instrument	Tiangen Biochemical Technology Co., Ltd
QT-1 vortex mixer	Shanghai Qite Analytical Instrument Co., Ltd
Mini-4LC miniature centrifuge	Zhuhai black horse medical instrument co., LTD
TD5A-WS bench top low speed centrifuge	Changsha Xiangyi Centrifuge Instrument Co. Ltd
DK-8D type electric thermostatic water tank	Shanghai Jinghong Experimental Equipment Co., Ltd
Gel imager	Shanghai Peiqing Technology Co., Ltd
FR-110 UV analysis device	Shanghai Furi Technology Co., Ltd
FR – 250 electrophoresis apparatus	Shanghai Furi Technology Co., Ltd
Multi-purpose horizontal electrophoresis tank	Beijing Baijing Biotechnology Co., Ltd
YXQ-LS-30II vertical pressure steam sterilizer	Shanghai Boxun Industrial Co., Ltd. Medical Equipment Factory
2720 Thermal Cycler	ABI
1–10 μl 12 pipettes	discovery
Ultra-clean table	Shanghai EPS Laboratory Equipment Co., Ltd
XW-80A vortex mixer	Shanghai Qite Analytical Instrument Co., Ltd
H1650-W table top micro high speed centrifuge	Changsha Xiangyi Centrifuge Instrument Co. Ltd
DHG-9053A type electric heating constant temperature air blowing drying oven	Shanghai Jinghong Experimental Equipment Co., Ltd
3730xl genetic analyze	ABI
Centrifuge5810R	Eppendorf, Germany
Milli-Q Academic	Millipore
Vc refrigerator BCD – 239	Henan Xinfei Electric Appliance Co. Ltd
HC-TP11-10 medical balance tray	Shanghai Precision Scientific Instrument Co., Ltd
Micro sampler	Eppendorf, Germany

#### 2.3.2 Main reagents

**Table:** 

Reagents	Company
Blood genomic DNA extraction kit	Tiangen Biochemical Technology Co., Ltd
Hotstar Taq	Qiagen
primer	Shanghai sangon
PCR reaction buffer	Takara
MgCl2	Takara
dNTP	GENERAY BIOTECH
PCR Marker	NEW ENGLAND Biolabs
agarose	BIOWEST
Ethidium bromide	Shanghai sangon
Bromophenol blue	Shanghai sangon
imLDR Multiplex	Shanghai Tianhao Biological Technology Co., Ltd
SAP	Promega
EXO-I	Epicenter
HI-DI	ABI
GeneScanTM-500	ABI

#### 2.3.3 Specimen collection

Fastening venous blood (3 ml) was collected from IMN patients and control group after morning, and the blood was anticoagulated with EDTA and frozen at −80°C.

#### 2.3.4 Experimental steps

(1) DNA extraction

Using the blood genome DNA extraction kit of Tiangen Company, the operation steps are as follows

Add 500 ~ 800 μl of whole blood sample to the centrifuge tube. Add 1 ml cell lysate CL and mix it upside down for 10 times. Centrifuge at 2000×g for 4 minutes and discard the supernatant. Add 400 μL cell lysate CL to resuspend the precipitation. Transfer the mixture to the sample tube. Place the sample tube in hole 4 of the T-frame of the TGuide M16 automatic nucleic acid extraction instrument, run the numbered program 102 (whole blood genomic DNA extraction procedure), select the sample volume of 400 μL and the final elution volume.

(2) SNP detection

Take 1 ul DNA, use 1% agarose electrophoresis to test its quality, and estimate its concentration, and then dilute the DNA to 5–10 ng/ μL according to the estimated concentration. Multiple PCR reaction:

PCR primers:

**Table:** 

SNP	Primer
rs2715918F	CACAAGAAAAGAGAGGCTCGATTTGA
rs2715918R	GAAACCCAAGATTCCGTTCTGGT
rs35771982F	GCATGGGAAATGCTGCTGTGTA
rs35771982 R	GAGCACATGAGCAGTAAAACAGTGG
rs3749117F	GCATGGGAAATGCTGCTGTGTA
rs3749117R	GAGCACATGAGCAGTAAAACAGTGG
rs2715928F	CATCCATCCCTGTGTCCACCTC
rs2715928R	TGGTGGTTCCTGCTTGGTTGTT
rs16844715F	CGATATATTAGTGGGGGACCATTTCAC
rs16844715R	GGATGGAGCATCCTGGTGAAAAC
rs4664308F	AGCCCTTCACTCGGAGGATCAC
rs4664308R	GGAGGCTTAATCAGGGGCAGGT
rs2187668F	CAACARTCATTTTACCACATGGTCCTC
rs2187668R	GKTGAAGAACAGGTAATTTGGGTTGATA

PCR conditions: The reaction system (total volume 20 l) consisted of 1 U Hotstartaq polymerase, 1X GC-I buffer,0.3 mM dNTP, 3.0 mM Mg2+,1 L multiplex PCR primer, and 1 μL sample DNA.

PCR cycle procedure: The reaction process is as follows: The temperature of 95°C 2 min, 94°C 20 s, 65°C 40 s, 72°C 1.5 min (repeated for 11 cycles, each cycle decreased by 0.5°C one after another), 94°C 20 s, 59°C 30 s, 72°C 1.5 min (repeated for 24 cycles), 72°C 2 min, 4°C forever.

Purification of multiplex PCR products: The 5 U SAP enzyme and 2 U Exonuclease I enzyme were added into the 20 l PCR product, which was first incubated at 37°C for 1 h and then inactivated at 75°C for 15 minutes.

Connection reaction:

Linking primers:

**Table:** 

SNP	Linking primers
rs2715918RG	TCTCTCGGGTCAATTCGTCCTTTCCGTTCTGGTACCAGTACGGTAAGTAC
rs2715918RA	TGTTCGTGGGCCGGATTAGTTCCGTTCTGGTACCAGTACGGTAAGCAT
rs2715918RP	GACTCTGGCAGCTCTTGAATTTCTAA
rs35771982FG	TTCCGCGTTCGGACTGATATGACCACTGCCAGCCAGCTTG
rs35771982FC	TACGGTTATTCGGGCTCCTGTGACCACTGCCAGCCAGCTTC
rs35771982FP	TTCATCCAGCTGATTGAGGCCTTTTTT
rs3749117FC	TCTCTCGGGTCAATTCGTCCTTCATCCAGCTGATTGAGGCCAAC
rs3749117FT	TGTTCGTGGGCCGGATTAGTCATCCAGCTGATTGAGGCCGAT
rs3749117FP	CCACACCTCCACTGTTTTACTGCTT
rs2715928FG	TCTCTCGGGTCAATTCGTCCTTCTTGAGGGCAGAGCACAGGAAGTG
rs2715928FA	TGTTCGTGGGCCGGATTAGTCTTGAGGGCAGAGCACAGGAAGTA
rs2715928FP	GCTAGCATGCTGTGAAGAATGTAACC
rs16844715RC	TTCCGCGTTCGGACTGATATGGAGCATCCTGGTGAAAACTTGG
rs16844715RT	TACGGTTATTCGGGCTCCTGTGGAGCATCCTGGTGAAAACTCGA
rs16844715RP	CATAGTCATTGCTAAAGGAAATGATCAGGT
rs4664308RG	TCTCTCGGGTCAATTCGTCCTTGGGGCAGGTAGAACAAGACCATTCTGAC
rs4664308RA	TGTTCGTGGGCCGGATTAGTGGGGCAGGTAGAACAAGACCATTCTGAT
rs4664308RP	TCTAAAGTGACCAAAGTCAGAGATGCTC T
rs2187668RC	TTCCGCGTTCGGACTGATATGTGACACAWATGAGGCAGCTGAGAGTCAG
rs2187668RT	TACGGTTATTCGGGCTCCTGTGTGACACAWATGAGGCAGCTGAGAGTCAA
rs2187668RP	TGAGGACCATGTGGTAAAATGAYTGTT

Connection reaction: The reaction system consisted of: high-temperature ligase 0.25 ul, 10X ligating buffer 1 ul, 5 ‘ligating primer mixture (1 μL) 0.4 μL, 3ʹ ligating primer mixture (2 μL) 0.4 ul, DDH2O 6 ul, purified multiplier PCR product 2 ul mixed.

Connection procedure: 4°C 1 min, 56°C 4 min (repeat 38 cycles), 4°C forever.

ABI3730XL sequencer on the connection product: The ligation products diluted with 0.5 ul were thoroughly mixed with 9 ul Hi-Di and 0.5 μl Liz500 Size Standard. After denaturation at 95°C for 5 minutes, the ligation products were detected by ABI3730XL sequencing instrument.

Use GeneMapper 4.1 (AppliedBiosystems, USA) to analyze the original data obtained from ABI3730XL sequencer.

#### 2.3.5 Statistical analysis

SPSS 19.0 statistical software was used for statistical analysis of the experimental data. The mean ± standard deviation (x ± s) was used to represent the measurement data conforming to the normal distribution. The t-test was used for comparison between groups. Counting data were compared by chi-square test. Population genetic Hardy-Weinberg equilibrium test was performed on genotypes by HWE software, and Bonferroni multiple comparison was used to correct the results of genetic data analysis. Chi-square test and Logistic regression were used to analyze the differences of allele and genotype frequency between the disease group and the control group, as well as the association between each SNP locus and IMN under different genetic models. Chi-square test and t test were used to analyze the relationship between different genotypes and the clinical manifestations of IMN. *P* < 0.05 indicates that the difference is statistically significant.

## 3. Results

### 3.1 Comparison of clinical characteristics between IMN group and healthy control group

The clinical baseline indexes of IMN patients and healthy control group were analyzed, and there was no statistically significant difference in age and BMI between the two groups (P > 0.05), gender, systolic blood pressure, diastolic blood pressure, SCR, ALB, CHOL and TG were statistically significant (P < 0.05), as shown in Table 2. As there was no abnormality in urine routine in the healthy control group, the 24-hour urine protein level was not detected.

**Table 2.** Clinical features of IMN group and control group

	IMN (*n* = 86)	Controls (*n* = 90)	*P*-value
Male/female	53/33	35/55	0.04
Age (year)	49 ± 13.21	48 ± 13.21	0.605
SBP (mmHg)	130.79 ± 18.55	123.58 ± 14.64	0.005
DBP (mmHg)	84.44 ± 10.63	77.63 ± 9.41	<0.001
BMI (kg/m^2^)	24.60 ± 4.53	23.49 ± 3.10	0.59
Scr (μmol/L)	77.14 ± 43.67	57.59 ± 14.21	<0.001
ALB (g/L)	23.53 ± 5.26	40.30 ± 5.0	<0.001
CHOL (mmol/L)	8.91 ± 3.29	4.01 ± 1.0	<0.001
TG (mmol/L)	3.08 ± 2.08	1.05 ± 0.69	<0.001

### 3.2 HWE (Hardy Weinberg Equilibrium) test

First, in order to conduct comparative detection of SNP gene in IMN patients, we divided the patients into groups according to pathological conditions. The basic data of the two groups were analyzed and compared to exclude the interference of redundant factors. The six SNPs of PLA2R rs16844715, rs2715918, rs2715928, rs355771982, rs3749117 and one SNP of HLA-DQA1 rs2187668 were tested by Hardy-Weiberg equilibrium. The genotyping of the 7 loci in the IMN group all met the Hardy-Weiberg balance test (P > 0.05), while the HLA-DQA1 rs2187668 did not meet the Hardy-Weiberg balance test in the healthy group (P < 0.05), so the SNP loci were removed in the subsequent studies. See Table 3.

**Table 3.** Hardy-Weinberg balance test

SNP	Major/minor		IMN genotype			HWE *P* value	Controls genotype			HWE *P* value
	(A/B)		AA	AB	BB		AA	AB	BB	
rs16844715	C/T	49		30	7	0.44	31	44	15	0.93
rs2187668	C/T	75		11	0	0.53	85	4	1	0.003
rs2715918	A/G	3		33	50	0.38	0	22	68	0.19
rs2715928	A/G	63		19	4	0.13	35	43	12	0.83
rs35771982	G/C	69		16	1	0.95	41	40	9	0.87
rs3749117	T/C	69		16	1	0.95	41	40	9	0.87
rs4664308	A/G	66		17	3	0.17	46	36	8	0.80

### 3.3 Distribution of alleles of each SNP locus in IMN group and healthy control group

The distribution of alleles of six SNP loci of PLA2R in IMN group and healthy control group was analyzed by chi-square test. The results showed that after Bonferroni correction, The distribution of alleles of rs16844715, rs2715928, rs35771982, rs3749117 and rs4664308 of SNP in IMN group and control group was statistically different (P* < 0.05). The allele distribution of rs2715918 was not statistically different between the IMN group and the control group (P* > 0.05). The C allele of rs16844715 (OR = 2.03, 95%CI: 1.29–3.19, P* = 0.0140) and the A allele of rs2715928 (OR = 3.18, 95%CI: 1.94–5.24, P* = 3.54E-5), G allele of rs35771982 (OR = 4.07, 95%CI: 2.34–7.08, P* = 4.96E-6), T allele of rs3749117 (OR = 4.07, 95%CI: 2.34–7.08, P* = 4.96E-6), the A allele of rs4664308 (OR = 2.63, 95%CI: 1.54–4.49, P* = 0.0028) is the risk gene of IMN, as shown in Table 4.

**Table 4.** Distribution of SNP locus alleles between IMN group and control group

SNP	Major/minor	Allele frequency in IMN		Allele frequency in Control		OR with 95% CI	Allele *P*	Adjusted P*
		Major	Minor	Major	Minor			
rs16844715	C/T	128 (0.74)	44 (0.26)	106 (0.59)	74 (0.41)	2.03 (1.29–3.19)	0.0020	0.0140
rs2715918	A/G	39 (0.23)	133 (0.77)	22 (0.12)	158 (0.88)	2.11 (1.20–3.70)	0.0096	0.0672
rs2715928	A/G	145 (0.84)	27 (0.16)	113 (0.63)	67 (0.37)	3.18 (1.94–5.24)	5.05E-6	3.54E-5
rs35771982	G/C	154 (0.90)	18 (0.10)	122 (0.68)	58 (0.32)	4.07 (2.34–7.08)	7.08E-7	4.96E-6
rs3749117	T/C	154 (0.90)	18 (0.10)	122 (0.68)	58 (0.32)	4.07 (2.34–7.08)	7.08E-7	4.96E-6
rs4664308	A/G	149 (0.87)	23 (0.13)	128 (0.71)	52 (0.29)	2.63 (1.54–4.49)	0.0004	0.0028

Note: * is corrected by Bonferroni.

### 3.4 Distribution of each SNP locus genotype in IMN group and healthy control group

We compared the genotype distribution of five SNP loci of PLA2R between IMN group and healthy control group, and corrected by Bonferroni. It was found that the distribution of each SNP rs2715928, rs35771982, rs3749117 and rs4664308 genotypes was statistically different between the IMN group and the healthy control group (P* < 0.05), as shown in Table 5.

**Table 5.** Distribution of SNP locus genotypes between IMN group and control group

SNP	Major/minor A/B	IMN (*n* = 86)			Controls (*n* = 90)			Genotype *P*	Adjusted P*
		AA (%)	AB (%)	BB (%)	AA (%)	AB (%)	BB (%)		
rs16844715	C/T	49 (57.0)	30 (34.9)	7 (8.1)	31 (34.4)	44 (48.9)	15 (16.7)	0.009	0.063
rs2715918	A/G	3 (3.5)	33 (38.4)	50 (58.1)	0 (0)	22 (24.4)	68 (75.6)	0.02	0.14
rs2715928	A/G	63 (73.3)	19 (22.1)	4 (4.6)	35 (38.9)	43 (47.8)	12 (13.3)	2.5E-5	1.75E-4
rs35771982	G/C	69 (80.2)	16 (18.6)	1 (1.2)	41 (45.6)	40 (44.4)	9 (10.0)	7.0E-6	4.9E-6
rs3749117	T/C	69 (80.2)	16 (18.6)	1 (1.2)	41 (45.6)	40 (44.4)	9 (10.0)	7.0E-6	4.9E-6
rs4664308	A/G	66 (76.7)	17 (19.8)	3 (3.5)	46 (51.1)	36 (40.0)	8 (8.9)	0.002	0.014

Note: * is corrected by Bonferroni.

### 3.5 Associations of each SNP locus with IMN susceptibility under different genetic models

By establishing additive model, dominant model and recessive model analysis, we found that, in the additive model, the three SNPs of PLA2R rs2715928 (OR = 5.40, 95%CI: 1.77–16.50, P* = 0.0217) and rs35771982 (OR = 15.15, 95%CI: 2.92–78.48, P* = 0.0084), rs3749117 (OR = 15.15, 95%CI: 2.92–78.48, P* = 0.0084) had a strong correlation with IMN. In the stealth model, homozygous gene risk type of the five SNPs, PLA2R rs16844715 (OR = 2.52, 95%CI: 1.38–4.61, P* = 0.0189) and rs2715928 (OR = 4.30, 95%CI: 2.31–8.03, P* = 3.14E-5), rs35771982 (OR = 4.85, 95%CI: 5.53–9.31, P* = 1.42E-5), rs3749117 (OR = 4.85, 95%CI: 5.53–9.31, P* = 1.42E-5) and rs4664308 (OR = 3.16, 95%CI: 1.67–5.97, P* = 0.0028) had a strong correlation with IMN, as shown in Table 6.

**Table 6.** Association of SNP loci with IMN susceptibility under different genetic models

Model		OR	95% CI	*P*	Adjusted P*
rs16844715					
Additive	CC/TT	3.39	1.28–8.96	0.0140	0.0980
	CT/TT	1.46	0.53–4.00	0.4605	3.2235
Dominant	(CC+CT)/TT	2.25	0.89–5.74	0.8730	6.1110
Recessive	CC/(CT+TT)	2.52	1.38–4.61	0.0027	0.0189
rs2715918					
Additive	AA/GG	–	–	0.0470	0.3290
	AG/GG	2.04	1.07–3.89	0.307	2.1490
Dominant	(AA+AG)/GG	2.23	1.18-.21	0.0140	0.0980
Recessive	AA/(AG+GG)	–	–	0.0739	0.5173
rs2715928					
Additive	AA/GG	5.40	1.77–16.50	0.0031	0.0217
	AG/GG	1.33	0.38–4.63	0.6588	4.6116
Dominant	(AA+AG)/GG	3.15	1.02–9.71	0.0452	0.3164
Recessive	AA/(AG+GG)	4.30	2.31–8.03	4.48E-6	3.14E-5
rs35771982					
Additive	GG/CC	15.15	2.92–78.48	0.0012	0.0084
	GC/CC	3.6	0.47–27.40	0.2161	1.5127
Dominant	(GG+GC)/CC	9.44	1.66–53.73	0.0114	0.0798
Recessive	GG/(GC+CC)	4.85	5.53–9.31	2.03E-6	1.42E-5
rs3749117					
Additive	TT/CC	15.15	2.92–78.48	0.0012	0.0084
	TC/CC	3.6	0.47–27.40	0.2161	1.5127
Dominant	(TT+TC)/CC	9.44	1.66–53.73	0.0114	0.0798
Recessive	TT/(TC+CC)	4.85	5.53–9.31	2.03E-6	1.42E-5
rs4664308					
Additive	AA/GG	3.83	1.04–14.08	0.0435	0.3045
	AG/GG	1.26	0.30–5.34	0.7545	5.2815
Dominant	(AA+AG)/GG	2.70	0.72–10.06	0.1390	0.9730
Recessive	AA/(AG+GG)	3.16	1.67–5.97	0.0004	0.0028

Note: * is corrected by Bonferroni.

### 3.6 Haplotype analysis of PLA2R gene

**Figure 1.**
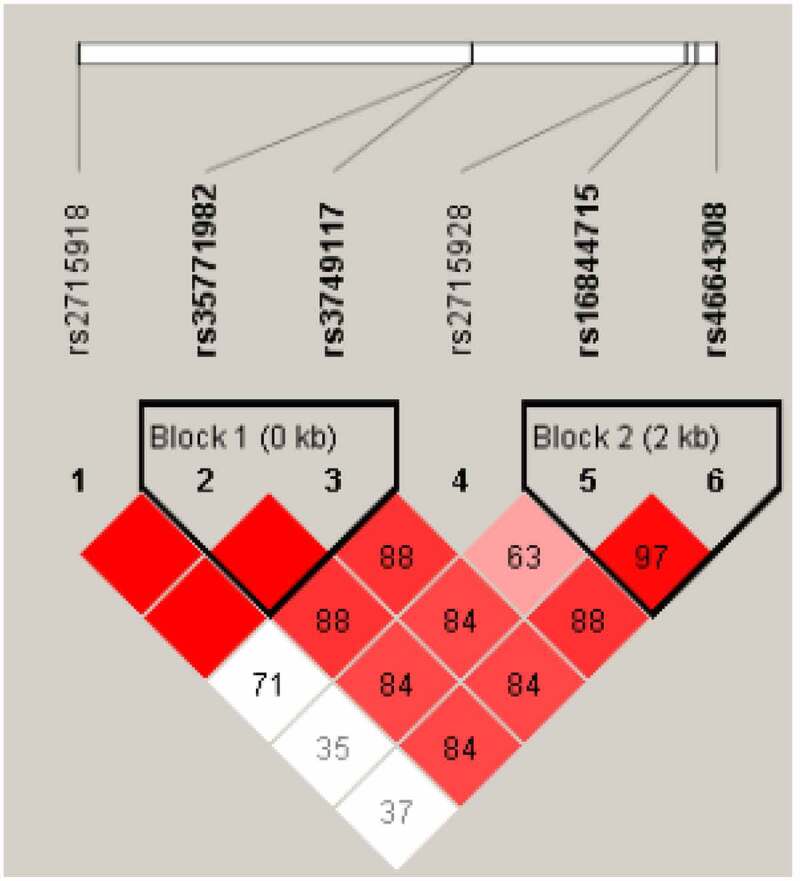
Linkage disequilibrium analysis of PLA2R gene

The linkage disequilibrium analysis of each locus of PLA2R gene was performed using Haploview software, and the linkage between each locus was found (D ‘> 0.8). Two blocks were obtained, Block1 was rs35771982 and rs3749117. Block2 is rs16844715 and rs4664308, see Figure 1. GT haplotype and CC haplotype can be obtained by building haplotype between genes of BLOCK1 and removing combinations less than 3% of frequency. Similarly, BLOCK2 can produce CA haplotypes, TG haplotypes and TA haplotypes. Chi-square test was used to analyze the distribution of each haplotype between the IMN group and the healthy control group. The results showed that the distribution of GT haplotype and CC haplotype in Block 1 was significantly different between the IMN group and the control group (P < 0.05). The distribution of CA haplotypes and TG haplotypes in BLOCK2 was significantly different between the IMN group and the control group (P < 0.05), as shown in Table 7.

**Table 7.** PLA2R haplotype analysis

Block	Haplotypes	Frequencies	Case ratios	Control ratios	*P*-value
Block 1	H1-GT	0.784	0.895	0.678	7.08E-7
	H2-CC	0.216	0.105	0.322	7.08E-7
Block 2	H3-CA	0.662	0.738	0.588	0.0030
	H4-TG	0.210	0.128	0.288	0.0002
	H5-TA	0.125	0.128	0.123	0.8776

### 3.7 Influence of interaction between various SNP sites of PLA2R gene on susceptibility to IMN

By analyzing the association between each SNP and IMN susceptibility, we found that the C allele of rs6844715, the A allele of rs2715928, the G allele of rs35771982, the T allele of rs3749117, and the A allele of rs4664308 were the disease risk genes of IMN. Therefore, we selected these five SNP loci, all located on PLA2R gene, to explore the interaction between each SNP and its association with IMN susceptibility.

**Figure 2.**
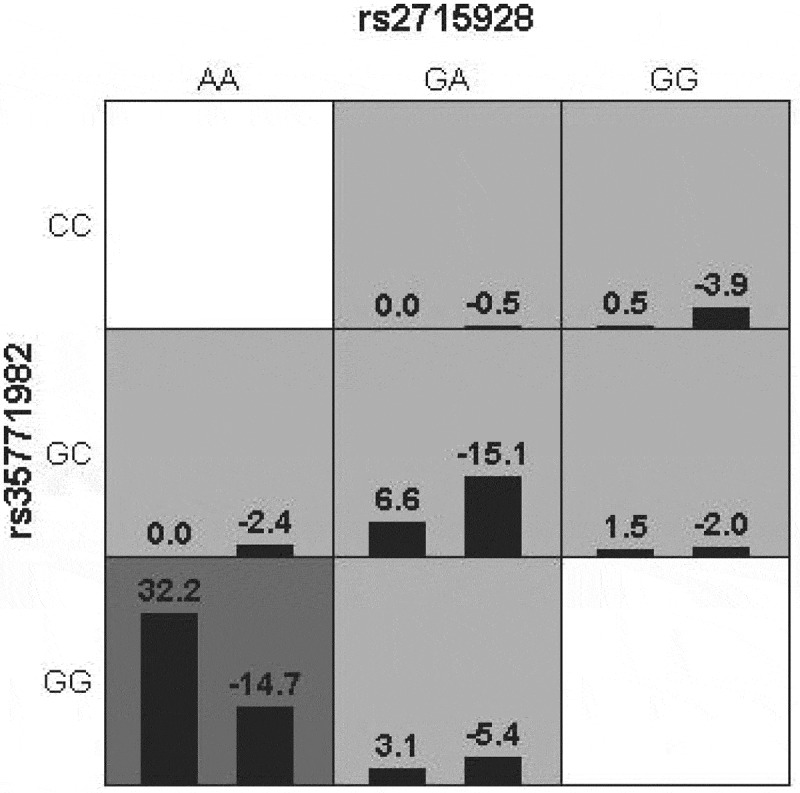
Combination of high-risk and low-risk genotypes under the best model

GMDR software was used to build a model to analyze the interaction between various SNP sites, and the results showed that the interaction model constituted by rs2715928 and rs35771982 sites was the best (Table 8). Through the different combinations of high-risk and low-risk genotypes, it can be seen that when the GG genotype of rs35771982 and the AA genotype of rs2715928 are combined, the color is the deepest, and the darker the color is, the greater the risk of the combination, so the combination genotype has the highest risk of disease, as shown in Figure 2.

**Table 8.** Establishing GMDR model to analyze the interaction between SNPs of PLA2R

Model	Training bal. acc.	Testing bal. acc.	CV consistency	Sign test (*P*)
rs355771982	0.6766	0.6514	6/10	10 (0.0010)
rs2715928, rs35771982	0.6998	0.6891	10/10	10 (0.0010)
rs2715928, rs35771982, rs4664308	0.7.64	0.6961	7/10	10 (0.0010)

### 3.8 Effects of different genotypes of PLA2R SNP sites on the clinical manifestations of IMN

We selected five SNP loci all located on PLA2R gene, rs6844715, rs2715928, rs35771982, rs3749117 and rs4664308, to explore the influence of different SNP genotypes on the clinical manifestations of IMN. The results showed that the ALB values of rs16844715 CT/CC genotype and TT genotype were different between the two groups (P < 0.05), but there was no statistical difference after Bonferroni correction. Because there was only one patient with rs35771982 CC genotype and rs3749117 CC genotype, we could not compare the effects of different genotypes of these two sites on the clinical manifestations of IMN. There was no statistical difference in clinical indicators between rs2715928 and rs4664308 genotypes, as shown in Tables 9 and Tables 10

**Table 9.** Effects of different genotypes on clinical manifestations of IMN

Clinical parameter	rs16844715			rs2715928			rs35771982		
	CT/CC	TT	*P*	GA/AA	GG	*P*	GC/GG	CC	*P*
Male/Female	48/31	5/2	0.58	48/33	4/0	0.10	53/33	1/0	0.42
Age (year)	49.68 ± 13.39	50.14 ± 11.88	0.93	49.67 ± 13.28	50.75 ± 13.53	0.87	49.89 ± 13.19	35	0.27
24 h proteinuria quantification (g)	6.30 ± 3.42	6.71 ± 2.97	0.76	6.39 ± 3.44	5.13 ± 0.90	0.06	6.33 ± 3.39	5.80	0.88
CHOL (mmol/L)	8.83 ± 3.38	9.84 ± 2.17	0.44	8.85 ± 3.36	10.20 ± 1.16	0.43	8.88 ± 3.30	11.93	0.36
TG (mmol/L)	3.14 ± 2.15	2.42 ± 1.03	0.38	3.11 ± 2.12	2.62 ± 1.50	0.65	3.08 ± 2.10	3.16	0.97
ALB (g/L)	23.90 ± 5.21	19.33 ± 4.17	0.03*	23.69 ± 5.22	20.38 ± 5.95	0.22	23.65 ± 5.18	13.5	0.06
Scr (μmol/L)	76.70 ± 44.62	82.26 ± 33.06	0.75	76.74 ± 44.64	85.60 ± 11.49	0.69	76.94 ± 43.88	95.30	0.68
BUN (mmol/L)	5.47 ± 2.04	6.69 ± 7.20	0.67	5.64 ± 2.80	4.19 ± 0.40	0.31	5.58 ± 2.77	4.61	0.73
BMI (kg/m^2^)	24.54 ± 4.63	25.37 ± 3.49	0.64	24.71 ± 3.79	22.40 ± 13.72	0.76	24.51 ± 4.48	32.40	0.08
eGFR (ml/min)	98.48 ± 24.71	93.05 ± 24.64	0.58	98.45 ± 25.11	89.50 ± 4.80	0.48	98.14 ± 24.73	89.00	0.71
SBP (mmHg)	131.51 ± 18.66	122.71 ± 16.31	0.23	131.01 ± 18.52	126.25 ± 21.45	0.62	130.96 ± 18.59	116.00	0.43
DBP (mmHg)	84.57 ± 10.92	83.00 ± 7.12	0.71	84.46 ± 10.35	84.00 ± 17.76	0.93	84.39 ± 10.69	89.00	0.67

Note: * is corrected by Bonferroni. P* = 0.36.

**Table 10.** Effects of different genotypes on clinical manifestations of IMN

Clinical parameter	rs3749117			rs4664308		
	TC/TT	CC	*P*	AG/AA	GG	*P*
Male/Female	52/33	1/0	0.43	50/33	3/0	0.16
Age (year)	49.89 ± 13.19	35.00	0.27	19.73 ± 13.29	49.33 ± 13.20	0.96
24 h proteinuria quantification (g)	6.34 ± 3.39	5.80	0.88	6.36 ± 3.43	5.56 ± 1.06	0.69
CHOL (mmol/L)	8.88 ± 3.30	11.93	0.36	8.81 ± 3.30	11.74 ± 1.99	0.13
TG (mmol/L)	3.08 ± 2.10	3.16	0.97	3.09 ± 2.12	2.87 ± 1.47	0.86
ALB (g/L)	23.65 ± 5.18	13.50	0.06	23.71 ± 5.20	18.67 ± 5.47	0.10
Scr (μmol/L)	76.94 ± 43.88	95.30	0.68	77.10 ± 44.39	78.67 ± 15.23	0.95
BUN (mmol/L)	5.58 ± 2.77	4.61	0.73	5.62 ± 2.79	4.16 ± 0.48	0.37
BMI (kg/m^2^)	24.51 ± 4.48	32.40	0.08	24.50 ± 4.53	27.43 ± 4.37	0.27
eGFR (ml/min)	98.14 ± 24.73	89.00	0.71	97.11 ± 25.01	96.11 ± 8.36	0.89
SBP (mmHg)	130.96 ± 18.59	116.00	0.43	131.27 ± 18.51	117.67 ± 17.56	0.21
DBP (mmHg)	84.39 ± 10.69	89.00	0.67	84.57 ± 10.70	71.00 ± 9.85	0.57

## 4. Discussion

It is well known that IMN is caused by the deposition of immune complexes formed by antigen–antibody binding in the subepithelium of the glomerulus, and the production of autoantibodies depends on the presentation of antigen to CD4 + T cells by MHC class II molecules. HLA-DQA1 consists of α and β chains, which are part of the heterodimer forming the antigen-binding channel. The MHC class II molecule is encoded by HLA gene, which is the most complex locus in the human genome. HLA-DQA1 is an HLA class II para-chain homologue gene, which spans 6246bp genomic DNA on chromosome 6p21 and encodes a protein of 255 amino acids.

SNP rs2187668 is located in the first intron of HLA-DQA1. The study of Stanescu and his colleagues shows that [11] the 282 SNPs in the HLA locus are associated with IMN, and the HLA-DQA1 allele is more associated with IMN susceptibility than the PLA2R allele in European Caucasians. HLA-DQA1 rs2187668 is also associated with IMN in Caucasian and African Americans in North America [12], Japanese [13], Indian [14] and Beijing, China [15]. However, in our study, HLA-DQA1 rs2187668 was excluded because it did not meet the Hardy-Weiberg equilibrium test in the healthy control group (P > 0.05). This may have something to do with the inadequate number of healthy controls we selected.

In addition, we also detected six SNPs located in PLA2R, among which rs2715928, rs16844715, rs35771982, rs3749117, and rs4664308 were significantly associated with IMN, while rs2715918 was not associated with IMN susceptibility.

PLA2R rs2715928 and rs16844715 are both located in the first intron of PLA2R. Thiri et al. [9] identified for the first time in their study that these two novel SNP loci are significantly associated with IMN susceptibility in the Japanese population. In addition, the A allele of rs2715928 and the C allele of rs16844715 are the risk genes of IMN, which are consistent with the results of our study.

PLA2R rs35771982 is located in the coding region of exon 5, C-type lectin domain 1 (CTLD1), where a mutation leads to the mutation of the 300th amino acid from histidine to aspartic acid. Several previous studies have shown that this SNP locus is significantly associated with IMN in Korea [7], Japan [13], Taiwan [8], European Caucasians [16], Beijing [15], North American Caucasians [12], and Indian population [14]. In addition to Korean population, The G allele of rs35771982 in the population of the above six regions was the risk gene of IMN, and our results were the same, while the C allele was the risk gene of IMN in the Korean population.

PLA2R rs3749117 is also located in the coding region of exon 5, C-type lectin domain 1 (CTLD1), and this SNP locus is significantly correlated with IMN in European Caucasites [16], Beijing [15], North American Caucasites and African Americans [12] with anti-PLA2R antibody positive, and Japanese [13]. Moreover, the T allele of rs3749117 is a risk gene of IMN, and our results are consistent with these data. In contrast, there is no association between rs3749117 and IMN in the Indian population [14].

PLA2R rs4664308 is located in the first intron of PLA2R, and GWAS confirmed that this SNP locus is closely associated with IMN susceptibility in European populations [11], and subsequently in Beijing [15], India [14], Spain [17], and Japan [18]. In addition, the A allele of RS rs4664308 is A risk gene of IMN, which is consistent with the results of our study.

PLA2R rs2715918 is located in the non-coding region of exon 16, which was previously found to be closely related to IMN in the European Caucasian [16] and Japanese population [13], but was not related to IMN in our study population.

Summerizing the above research results, we found that the same SNP loci were not fully expressed in different regions, different populations and different races, and even the risk genes of diseases were different, indicating that gene polymorphism and disease susceptibility were affected by population, region, race and environment [19].

PLA2R belongs to the mannose receptor family and is a type I transmembrane glycoprotein. It consists of a large extracellular segment, which includes an N-terminal cysteine-rich region, a fiberectin type II domain, a tandem repeat of eight C-terminal lectin domains (CTLD), a transmembrane domain, and a short intracellular C-terminal region. The gene encoding PLA2R is located in the region 2q23 ~ q24 of human chromosome, and the mutation of this gene can affect the production of amino acids. For example, the mutation of SNP rs35771982 G allelic gene located in exon 5 region CTLD1 causes the mutation of the 300th amino acid from histidine to aspartic acid. CTLD is involved in several functions such as extracellular matrix organization, endocytosis, complement activation, pathogen recognition, and cell–cell interaction, and changes in the structure of these regions may affect these important functions.

The study of Coenen and colleagues showed [16] that although IMN is a rare disease, its rarity may not be the result of rare gene mutations in PLA2R, so the research focus has been turned to the gene interaction between HLA and PLA2R. Stanescu and his colleagues found that [11] HLA-DQA1 rs2187668 and HLA-DQA1 rs2187668 are associated with a 78.5-fold increased risk of disease in homozygous genes. Although it is not clear how the HLA risk allele affects the epitope presentation of HLA class II molecules in IMN, one possibility is that the antigen is presented to T cells to initiate T cells to assist in the production of anti-PLAP2R antibodies. These risk alleles encode protein receptors that interact with each other during antigen presentation to stimulate T cells. In this case, the PLA2R protein processed in macrophages or dendritic cells appears on the cell surface as a PLA2R peptide bound to the HLA-DQA1 antigen-binding channel. The genetic variation in HLA-DQA1 can alter its conformation and thus control the shape of the antigen-binding groove, thereby altering the specificity of immunogen presentation. Mutations in the PLA2R gene can control the possible enzymatic cleavage pattern of the PLA2R by altering the amino acid that produces or destroys the cleavage site, as well as by changing the splicing site or the transcription level that can be used to fragment the protein species, leading to higher levels of antigenic peptide expression [20].

In conclusion, the development of IMN and its rarity in the general population may not be the result of rare genetic mutations in PLA2R, but rather a rare combination of three relatively common conditions: HLA-DQA1 confers susceptibility to autoimmunity, and polymorphisms in PLA2R produce a unique conformation identified by HLA class II on antigen-presenting cells and, as a target of the autoantibodies, produce IgG4 anti-PLA2R antibodies that activate the complement’s lectin pathway and cause podocyte damage and proteinuria [21].

However, in our study, we found that there were also high-risk genes in the control population, suggesting that other risk factors should be involved in the development of IMN. A study from China pointed out that the increased incidence of IMN is related to long-term exposure to high concentrations of PM2.5 [19], so environmental factors are also involved in the incidence of IMN, but the pathogenesis is still unclear. IMN may be triggered when three independent risk factors, namely genetic polymorphisms in the PLA2R and HLA regions and environmental factors, combine.

Due to the great differences in clinical manifestations, course of disease and prognosis among patients with different IMN [22], we analyzed the influence of different genotypes of PLA2R SNPs on the clinical manifestations of IMN. There were differences in ALB values between rs16844715 CT/CC genotype and TT genotype groups (P < 0.05), but there was no statistical difference after Bonferroni correction. There was no difference in clinical indicators between rs2715928 and rs4664308 genotypes. Because there was only one rs35771982 CC genotype and only one rs3749117 CC genotype, we could not conduct statistical analysis on these two loci. A study from Taiwan, China [18] pointed out that rs6757188 and rs35771982 polymorphisms were not associated with different clinical manifestations of IMN patients, but were associated with low remission rate after treatment. Studies in Beijing, China [15] have shown that the risk genotype of PLA2R is closely related to the pathological phenotype of IMN. Studies in Spain have shown that immunosuppressive therapy is more effective in patients with a combination of IMN susceptibility genotypes (HLA-DQA1 rs2187668 AA/AG and PLA2R rs4664308 AA) and slow progression of renal function.

This study also has some limitations. First of all, due to the small sample size, the SNP loci confirmed in previous studies did not meet the Hardy-Weiberg equilibrium test in our population and were removed. In addition, the number of patients with protective genotypes of individual loci was too small for statistical analysis. Secondly, there were differences in gender distribution between the IMN group and the healthy control group, which may affect the results of gender distribution of different genotypes. Finally, our findings are limited to risk loci and causal variants of the disease. In order to apply this genotype information to clinical management, it is necessary to explore further correlations with disease phenotypes, such as severity of onset, anti-PLA2R antibody titer, response to immunosuppressive therapy, and long-term renal prognosis.

## 5. Conclusion

PLA2R rs2715928 and rs16844715 are associated with susceptibility to IMN. The C allele of rs16844715, the A allele of rs2715928, the G allele of rs35771982, the T allele of rs3749117, and the A allele of rs4664308 are the dangerous genes of IMN. The combination of GG genotype at rs35771982 and AA genotype at rs2715928 poses the greatest risk of disease. Haplotype may affect susceptibility to IMN. The risk genotype had no effect on the clinical manifestations of IMN.
